# N6-methyladenosine (m6A)-mediated up-regulation of long noncoding RNA LINC01320 promotes the proliferation, migration, and invasion of gastric cancer via miR495-5p/RAB19 axis

**DOI:** 10.1080/21655979.2021.1953210

**Published:** 2021-07-21

**Authors:** Naijun Hu, Hong Ji

**Affiliations:** a Department of Obstetrics and Gynecology, The First Affiliated Hospital of Jinzhou Medical University, Jinzhou, China; b Department of General Surgery, The First Affiliated Hospital of Jinzhou Medical University, Jinzhou, China

**Keywords:** LncRNA linc01320, miR-495-5p, gastric cancer, n6-methyladenosine, rab19

## Abstract

Gastric cancer is one of the most common malignant tumors. Long non-coding RNAs play crucial roles in gastric cancer progression. This study investigated the effect of LINC01320 on malignant behaviors of gastric cancer cells and explored its possible molecular mechanism. LINC01320 expression in gastric cancer tissues and cell lines was measured by qRT-PCR. Cell proliferation, transwell, and cell cloning assays were used to detect the effect of LINC01320 on the proliferation, migration, and invasion abilities, respectively, of gastric cancer cells. Bioinformatics analysis was used to predict the binding of miR-495-5p with LINC01320 and RAB19. A luciferase reporter assay was performed to verify their interactions. Finally, the N^6^-methyladenosine (m6A) modification of LINC01320 by METTL14 was identified through RIP experiments. LINC01320 was highly expressed in gastric cancer tissues and cells. LINC01320 overexpression promoted the proliferation, migration, and invasion of gastric cancer cells, while LINC01320 knockdown exerted the opposite effects. Moreover, miR-495-5p was predicted and demonstrated to target LINC01320 and RAB19. LINC01320 sponged miR-495-5p to regulate the expression of RAB19. Additionally, LINC01320-induced increases in cell viability, migration, and invasion of gastric cancer were alleviated by miR-495-5p and silenced RAB19. Furthermore, epigenetic studies showed that METTL14-mediated m6A modification led to LINC01320 up-regulation. METTL14 regulated the m6A modification of LINC01320. Overexpressed LINC01320 contributed to the aggressive phenotype of gastric cancer cells via regulating the miR-495-5p/RAB19 axis. This finding may provide new potential targets for treating gastric cancer.

## Graphical Abtstract

**Figure:**
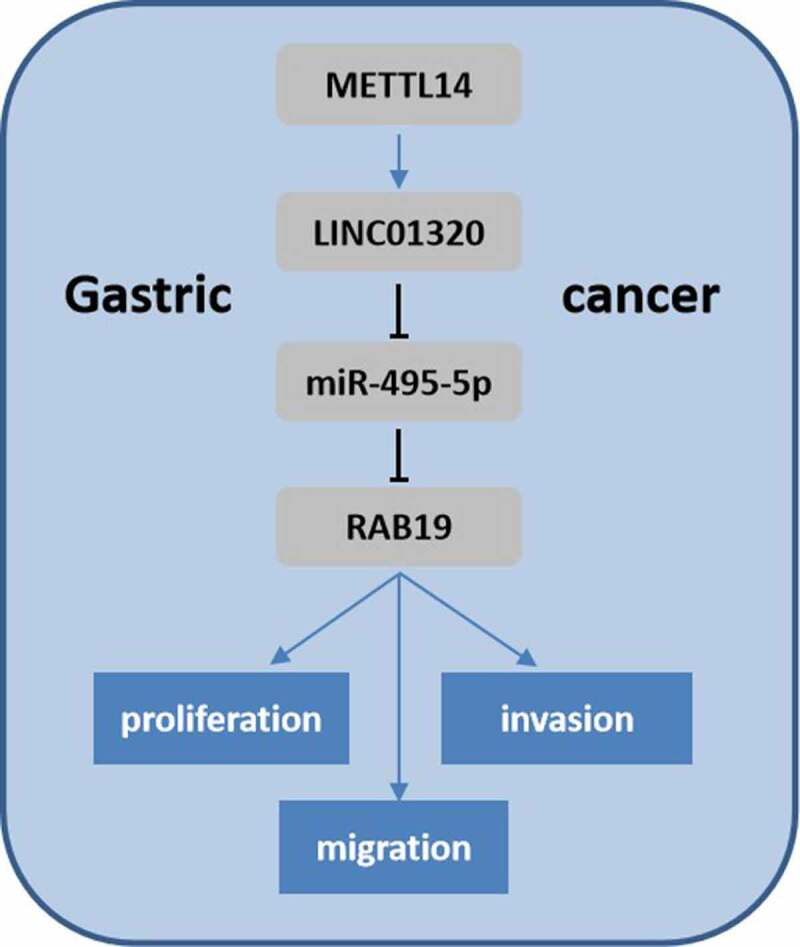

## Introduction

Gastric cancer is the third leading cause of cancer-related death, characterized by high metastasis and high invasiveness. The incidence of gastric cancer is relatively insidious, and most gastric cancer patients are diagnosed at an advanced stage [1]. In addition, the occurrence and development of gastric cancer are affected by epigenetics [2]. Thence, investigation of the molecular mechanisms underlying the development of gastric cancer is of vital importance.

Long non-coding RNAs (lncRNAs) are a group of non-coding RNAs with a length of more than 200 nucleotides [3]. lncRNAs modulate gene expression at transcriptional or posttranscriptional levels and in physiological processes, including proliferation, differentiation, and necrosis [4]. Studies have shown dysregulated expression of numerous lncRNAs, indicating their critical roles in tumor progression [5–7]. However, the effect and mechanism of LINC01320 on gastric cancer are still unclear.

MicroRNAs (miRs) are a type of non-coding RNA with the length of about 22 nucleotides [8]. Numerous miRs have been found to be abnormally expressed in tumor cells and play important roles in tumor cell proliferation, differentiation, apoptosis, and metastasis [9,10]. Nevertheless, the effect of miR-495-5p in gastric cancer remains unclear.

N^6^-methyladenosine (m6A) RNA modification is the most abundant endogenous apparent transcriptional modification of mRNAs, which is mediated by the m6A methyltransferase complex [11]. Recently, numerous studies have indicated that m6A modification is involved in the dysregulated expression of lncRNAs [12,13]. For instance, m6A-induced lncRNA RP11 triggers the dissemination of colorectal cancer cells via up-regulation of Zeb1.

In the present study, we supposed that the dysregulated LINC01320 must play critical roles in the development of gastric cancer. Thus, the aim of this study was to elucidate the function of LINC01320 on gastric cancer progression and the underlying mechanism. We found that LINC01320 can promote the proliferation, migration, and invasion of gastric cancer cells by sponging miR-495-5p. We further determined that RAB19 is a target of miR-495-5p, which targets RAB19 to inhibit its expression. Mechanistically, METTL14-mediated m6A modification induces the up-regulation of LINC01320, which subsequently affects gastric cancer progression through the miR-495-5p/RAB19 axis. Our data provide new insights into lncRNAs in gastric cancer.

## Materials and methods

### Clinical specimen

The clinical samples of gastric cancer patients, including cancer tissues and paired normal tissues, were collected from The First Affiliated Hospital of Jinzhou Medical University. The present study was approved by the Ethics Committee of The First Affiliated Hospital of Jinzhou Medical University. Written informed consent was obtained from the patients.

### Cell culture

Human gastric cancer cells MKN7, MNK45, AGS, HGC27, NCI-N87 and human gastric mucosal cell line GES-1 were purchased from American Type Culture Collection (Manassas, VA, USA). Fetal bovine serum, trypsin, and medium were purchased from Gibco (Wellesley Hills, MA, USA). The cells were cultured in Dulbecco’s modified Eagle’s medium (DMEM) containing 10% fetal bovine serum (FBS), 100 g/mL streptomycin, and 100 U/mL penicillin in an incubator at 37°C and 5% CO_2_. The solution was changed every 2 days, and when the cell growth and fusion degree reached more than 85%, 0.25% trypsin was used for cell digestion and passage [14].

### Cell transfection

Gastric cancer cells were inoculated into 6-well cell culture plates at a concentration of 2 × 10^5^ cells per well. When the cell growth density reached more than 70%, plasmids were transfected using the Invitrogen Lipofectamine 2000 transfection kit (Life Technologies, Carlsbad, CA, USA) according to the manufacturer’s instructions [15].

### Cell proliferation assay

WST-1 (Beyotime, Shanghai, China) was used to determine the proliferation of gastric cancer cells in each group. AGS and HGC27 cells in each group were collected 24 h after transfection. After washing with phosphate-buffered saline (PBS) and digestion with trypsin, cells were resuspended with complete medium. Then, cells were inoculated into 96-well plates at a concentration of 2 × 10^3^ cells per well. The cells were cultured in an incubator for 24, 48, and 72 h. Each well was incubated with WST-1 solution for 2 h, and the absorbance (OD) value of cells in each well was determined at 450 nm [16].

### Cell clone formation experiment

Gastric cancer cells were digested and re-suspended in DMEM containing 10% FBS. Then, the cell suspension (1000 cells/mL) was seeded onto 6 cm culture dishes. After culturing in an incubator for 2–3 weeks, the supernatant was discarded and cells were washed with PBS for 3 times. After fixing with 4% paraformaldehyde, the cells were stained with GIMSA for 20 min. The colonies containing more than 50 cells were counted under a microscope (Leica, Wetzlar, Germany) [17].

### Transwell assay

At 24 h after transfection, gastric cancer cells were digested and re-suspended in DMEM without FBS. Cells (5 × 10^5^ cells/mL) were added to the upper transwell chamber. A complete medium containing 12% FBS was added to the lower chamber. After 24 h of culture, cells on the lower surface were fixed with methanol and then stained with 0.1% crystal violet for 30 min. Finally, the number of invaded cells was counted under a microscope (Nikon, Tokyo, Japan). To detect invasion, Matrigel (BD Biosciences, San Jose, CA, USA) was first used to pre-coat the upper membrane. The other procedures are as the same as those used to detect migration [18].

### Double luciferase reporter assay

Wild-type or mutant fragments of RAB19 or LINC01320 containing the predicted binding sites were cloned into the GV272 vector using mRNA binding. After verification by DNA sequencing, miR-495-5p mimics or control plasmids were transfected into cells using Lipofectamine 2000 according to the manufacturer’s instructions. After transfection for 48 h, cells were collected and analyzed using a dual-luciferase reporting system [19].

### RNA pull down

miR-495-5p sequences were ethelized by Biotin RNA Labeling Mix. After DNA digestion by Rnas I, they were purified using the RNeasy Mini Kit (Qiagen, Valencia, CA, USA). RNA structure buffer (10 mmol/L Tris pH = 7, 0.1 mol/L KCl, 10 mmol/L MgCl_2_) was heated to 95°C with 1 μg labeled RNA. After 2 min, RNA was incubated on ice for 3 min and then allowed to rest at room temperature for 30 min to form an appropriate secondary structure. AGS and HGC27 cells were collected, and cell lysates (Sigma, St. Louis, MO, USA) were added for 1 h at 4 °C. The lysate was centrifuged, and the supernatant was collected and transferred to an RNase-free centrifuge tube. Streptavidin agarose beads (50 μL) were added, and samples were incubated for 1 h. The eluent was collected for RT-PCR detection of the target gene [20].

### qRT-PCR

The total RNA in the cells after transfection was extracted using Trizol regent according to the manufacturer’s instructions. Thereafter, the total RNA was reverse-transcribed into cDNA using a reverse transcription kit (provided by Shanghai Sangon Biological Engineering Co., LTD., Shanghai, China). The reaction was terminated at 4°C. Three replicates were set for each sample, and 2^−ΔΔCt^ was used for relative quantitative analysis of the data [21].

### Statistical analysis

SPSS 20.0 (SPSS Inc., Chicago, IL, USA) statistical software was used for data analysis, and measurement data were expressed as mean ± SD.

## Results

As LINC01320 is up-regulated in gastric cancer tissues and cell lines, we supposed that LINC01320 should play critical roles in the development of gastric cancer. Thus, the aim of this study was to elucidate the function of LINC01320 on gastric cancer progression and the underlying mechanism. Function studies including WST-1 and transwell assays were used to detect the proliferation and migration of gastric cancer cell. Luciferase and RNA pull down assays were performed to detect the interaction between miR-495-5p and its target genes. In addition, we tried to explore the up-stream regulation of LINC0130 and found that METTL14 mediated m6A modification induces the up-regulation of LINC01320.

### LINC01320 expression is up-regulated in gastric cancer

**Figure 1.**
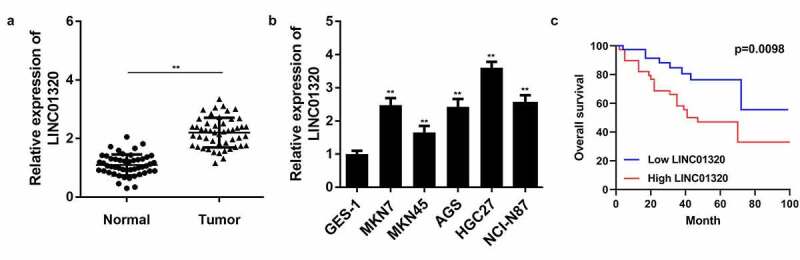
**LINC01320 is overexpressed in gastric cancer tissues and cells**. (a) The expression of LINC01320 in tumor tissues. (b) The expression of LINC01320 expression in normal gastric mucosal cell line and gastric cancer cell lines. (c) Kaplan-Meier curve showed the overall survival in gastric cancer patients according to LINC01320 expression. Red curve represents patients with high LINC01320 expression, while blue curve represents low LINC01320 expression according to the median value of LINC01320. **P< 0.01 vs healthy control or QSG7701

To study the role of LINC01320 in gastric cancer, we first analyzed LINC01320 expression in gastric cancer tissues and cells using qPCR. The results showed that LINC01320 was significantly up-regulated in gastric cancer compared with adjacent control tissues (Figure 1a). Further, LINC01320 was significantly overexpressed in gastric cancer cells (Figure 1b). In addition, Kaplan-Meier analysis showed that gastric cancer patients with high LINC01320 expression had lower overall survival rate than those with low LOC285194 expression (Figure 1c).

### LINC01320 promotes the malignant behavior of gastric cancer cells

**Figure 2.**
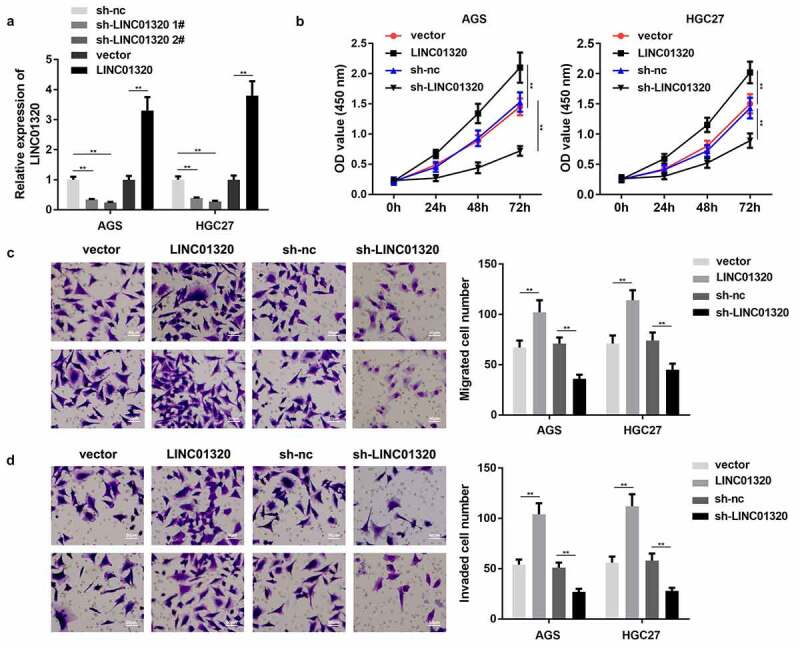
**Overexpression of LINC01320 promotes the malignant progression of gastric cancer**. (a) Detection of LINC01320 transfection efficiency. (b) After transfected with LINC01320 overexpression or knockdown plasmids, the proliferation ability of gastric cancer cells AGS and HGC27 was detected. (c) After transfected with LINC01320 overexpression or knockdown plasmids,, the migration ability of gastric cancer cells AGS and HGC27 was detected. (d) After transfected with LINC01320 overexpression or knockdown plasmids,, the invasive ability of gastric cancer cells AGS and HGC27 was detected. **P< 0.01 vs sh-nc or vector

We next studied the effects of LINC01320 overexpression or knockdown on the proliferation, invasion, and migration of gastric cancer cells. First, we tested the efficiency of the LINC01320-overexpressing vector and shRNA. LINC01320 expression was significantly increased by LINC01320 overexpression plasmids and decreased by LINC01320 knockdown plasmids, suggesting that cells were efficiently transfected (Figure 2a). Furthermore, LINC01320 promoted the viability of AGS and HGC27 cells while LINC01320 knockdown conferred the opposite effects (Figure 2b). Moreover, the migration and invasion abilities of gastric cells were enhanced by LINC01320 overexpression and suppressed by LINC01320 knockdown (Figure 2c, d).

### LINC01320 targets miR-495-5p in gastric cancer cells

**Figure 3.**
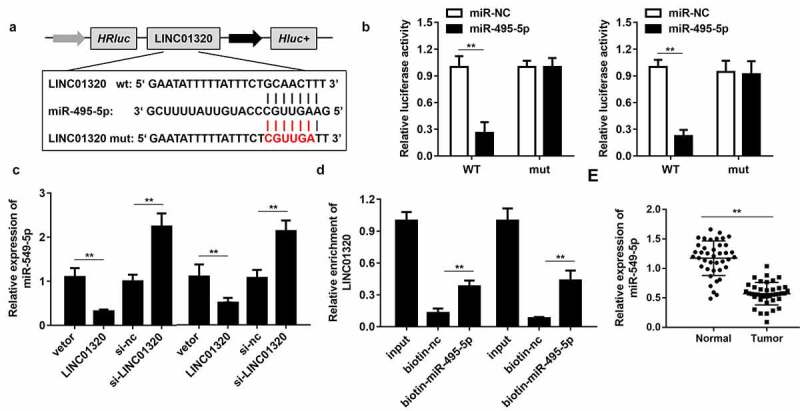
**LINC01320 sponges miR-495-5p in cancer cells**. (a) The binding sites between LINC01320 and miR-495-5p. (b) Dual luciferase reporter gene detection experiment to verify whether LINC01320 could bind to miR-495-5p. (c) Detection of miR-495-5p expression level. (d) RNA pulled down confirmed the interaction between LINC01320 and miR-495-5p. (e) Detection of miR-495-5p expression in gastric cancer tissues and adjacent tissues. **P< 0.01 vs miR-nc, vector, biotin-nc, normal tissues

To further study the mechanism of LINC01320 in gastric cancer, we analyzed the downstream miRNAs associated with LINC01320. The predicted results indicated that LINC01320 can bind to miR-495-5p (Figure 3a). Dual-luciferase reporter gene detection further confirmed the binding between LINC01320 and miR-495-5p (Figure 3b). The expression of miR-495-5p was inhibited by LINC01320 overexpression and up-regulated by LINC01320 knockdown (Figure 3c). The RNA pull-down assay showed that the miR-495-5p probe effectively enriched LINC01320 (Figure 3d). Further detection determined that miR-495-5p was down-regulated in gastric cancer tissues (Figure 3e). The above experimental results indicate that miR-495-5p can be sponged by LINC01320 and may play an important role in the malignant behavior of gastric cancer.

### RAB19 is the target gene of miR-495-5p

**Figure 4.**
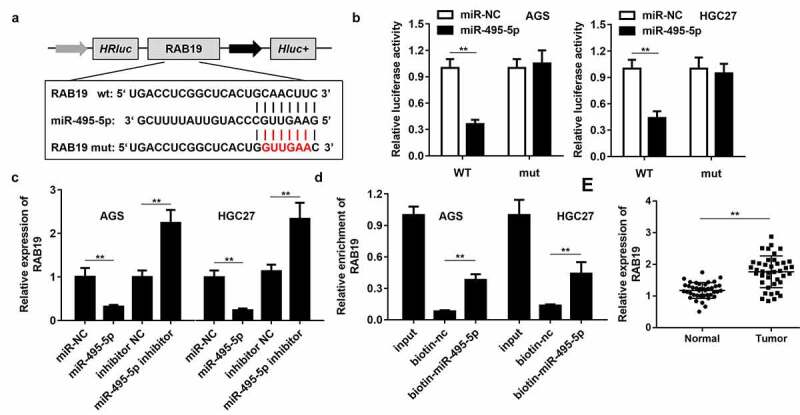
**RAB19 is the target gene of miR-495-5p**. (a) The binding sites between RAB19 and miR-495-5p. (b) Dual luciferase reporter gene detection experiment to verify whether RAB19 could bind to miR-495-5p. (c) Detection of RAB19 expression level. (d) RNA pulled down confirmed the interaction between RAB19 and miR-495-5p. (e) Detection of RAB19 expression in gastric cancer tissues and adjacent tissues. **P< 0.01 vs miR-nc, vector, biotin-nc, normal tissues

To study the downstream regulatory target genes of miR-495-5p, we used bioinformatics tools to predict the target of miR-495-5p, and RAB19 was selected for further analysis. Figure 4a displays the binding sites of miR-495-5p and RAB19. The binding of miR-495-5p with RAB19 was confirmed by dual-luciferase reporter assay (Figure 4b). Overexpression of miR-495-5p inhibited the expression of RAB19, while inhibition of miR-495-5p up-regulated RAB19 (Figure 4c). The RNA pull-down assay confirmed the interaction between miR-495-5p and RAB19 (Figure 4d). Further test results showed that RAB19 expression was up-regulated in gastric cancer tissues (Figure 4e).

### LINC01320 promotes the cell proliferation, migration, and invasion of gastric cells via regulating the miR-495-5p/RAB19 axis

**Figure 5.**
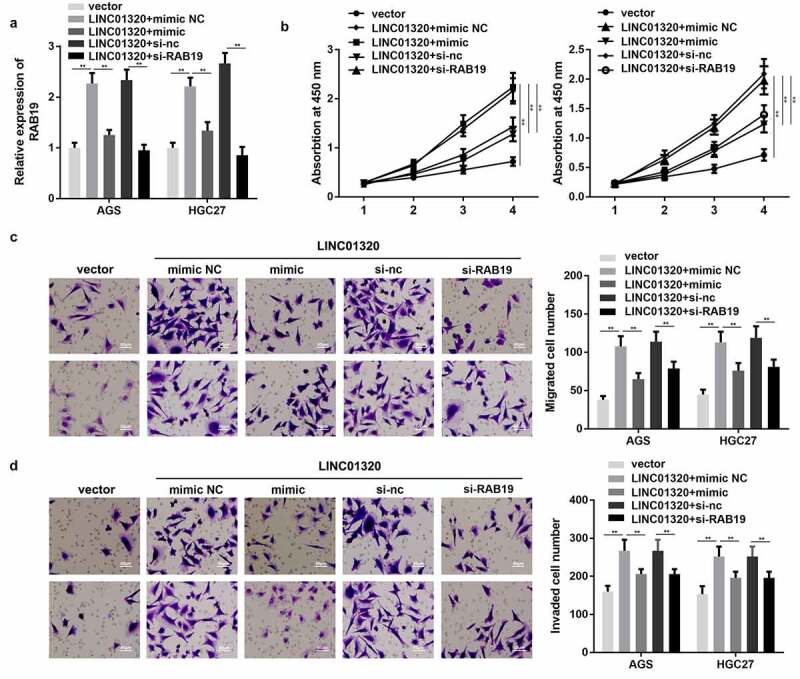
**MiR-495-5p overexpression or knockdown of RAB19 reversed the effect of LINC01320 on the proliferation, migration, and invasion of gastric cancer cells**. (a) Expression of RAB19 was detected. (b) Cell proliferation experiments after treatment in different groups. (c) Cell migration ability test after different treatments. (d) Cell invasion ability tests after different treatments. **P< 0.01 vs sh-nc, ^##^P < 0.01 vs sh-LINC01320 + inhibitor nc or sh-LINC01320 + vector

To further confirm the interaction between miR-495-5p and LINC01320 or between RAB19 and LINC01320, we performed rescue experiments. As shown in Figure 5a, the expression of RAB19 was significantly increased by overexpression of LINC01320, while this was reversed by up-regulation of miR-495-5p or knockdown of RAB19. Additionally, overexpression of miR-495-5p or silencing of RAB19 both reversed the effect of LINC01320 on the proliferation, migration, and invasion of gastric cells (Figure 5b-d).

### LINC01320 is regulated by m6A modification of METTL14

**Figure 6.**
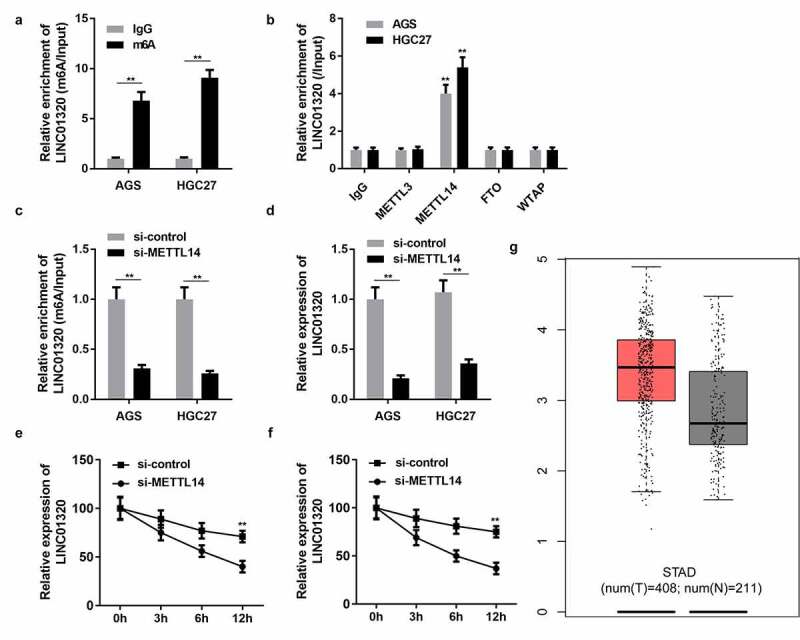
**LINC01320 is regulated by m6A modification of METTL14**. (a) Protein immunoprecipitation assay to detect the m6A modification of LINC01320. (b) RIP antibody test of different M6A-related proteins. (c) METTL14 interference efficiency detection. (d) LINC01320 expression was detected by qPCR after METTL14 interference. (e and f) METTL14 regulates the m6A modification of LINC01320. **P< 0.01 vs IgG, si-control, or healthy control

Epigenetic modifications play an important role in gastric cancer progression. To elucidate the regulatory mechanism of LINC01320 expression, we analyzed whether LINC01320 is modified with m6A. M6A antibody and control IgG antibody were used to conduct a pull-down assay. The assay results revealed that LINC01320 expression in the m6A group was significantly higher than that in the IgG group (Figure 6a). Many enzymes can regulate m6A modification in cells. To determine which m6A enzyme regulates LINC01320 modification, we used antibodies of different m6A-related proteins to conduct a RIP experiment and detect the expression of LINC01320 in the pulled-down products. The experimental results showed that METTL14 and FTO significantly promoted the enrichment of LINC01320, indicating that METTL14 and FTO were involved in the m6A modification of LINC01320 (Figure 6b). Furthermore, knockdown of METTL14 decreased the enrichment of LINC01320, and METTL14 silencing reduced LINC01320 RNA stability (Figure 6e and f). Bioinformatics analysis revealed that LINC01320 was overexpressed in gastric cancer (Figure 6g).

## Discussion

Dysregulated lncRNAs function as tumor suppressors or promoters in the occurrence and development of tumors [22]. lncRNAs modulate gastric cancer progression via regulating proliferation, metastasis, invasion, and other biological processes [23–25]. For instance, Ren et al. revealed that TUG1 sponges miR-145 to promote the development of gastric cancer by reducing the inhibitory effect of miRNA-145 on gastric cancer development [26]. The up-regulation of the lncRNA highly up-regulated in liver cancer (HULC) contributes to the occurrence and development of gastric cancer via regulating miR-372/CAMP-responsive element-binding protein. However, the potential roles of LINC01320 in gastric cancer are unknown. Previously, differential expression of LINC01320 was reported in endometrial and ovarian cancer cells [27]. LINC01320 may be involved in endometrial maturation [28]. In this study, LINC01320 was down-regulated in gastric cancer tissues and cells. Moreover, overexpression of LINC01320 promoted the proliferation, invasion, and migration of gastric cancer cells, while knockdown of LINC01320 exerted the opposite effects. These results indicate that LINC01320 may act as a tumor promoter in gastric cancer. However, the underlying mechanisms remain unclear.

The regulatory mechanisms of lncRNAs are complicated. To elucidate the mechanism of LINC01320 in gastric cancer progression, we focused on its effect as an miRNA sponge. It was found that LINC01320 can directly bind with miR-495-5p. miR-495-5p has been reported to play critical roles in the progression of ovarian cancer and alleviated lnc00908-induced ovarian cancer development [9]. In this study, miR-495-5p was demonstrated to be down-regulated in gastric cancer tissues. To our knowledge, this is the first study to unveil the potential roles of miR-495-5p in gastric cancer.

It is well known that miRNAs bind with their target genes to degrade the level of gene expression [29–31]. RAB19 emerged in metazoans as an expansion from RAB19, and humans have two paralogs, RAB19 and RAB43 [32–34]. It was reported that RAB19 is negatively correlated with the tumor purity of oral squamous cell carcinoma and positively correlated with the immune infiltration of B cells, CD8 + T cells, CD4 + T cells, macrophages, neutrophils, and dendritic cells [35]. However, the roles of RAB19 in gastric cancer have not been elucidated. This study found that RAB19 was significantly up-regulated in gastric cancer, suggesting that it may function as an oncogene in gastric cancer. Further study showed that RAB19 expression was increased by LINC01320 and decreased by miR-495-5p. Moreover, down-regulation of RAB19 antagonized the aggressive behaviors of gastric cancer induced by LINC01320 overexpression. These findings extend our understanding of the role of RAB19 in the carcinogenesis of gastric cancer.

The methyltransferase catalyzes the production of m6A in the form of complexes in the nucleus. The complex consists of an interacting protein, such as METTL14 and WTAP [36]. These proteins modulate m6A modification in non-coding RNAs. For instance, the lncRNA KCNK15-AS1 inhibits the metastasis of pancreatic cancer through ALKBH5 demethylation [37]. Moreover, it was also found that m6A was distributed differently in mRNA and lncRNA, indicating that m6A modification of lncRNA may differ from that of mRNA, and its specific mechanism needs further study [38,39]. In this study, METTL14 was involved in the m6A modification of LINC01320 and induced the up-regulation of LINC01320. Hence, the METTL14/LINC01320/miR-495-5p/RAB19 axis may play a positive role in the development of gastric cancer.

However, this study has some limitations. The effect of the LINC01320/miR-495-5p/RAB19 axis on gastric cancer has not been studied at the animal level. Only METTL14, a methyltransferase, was found to mediate the m6A modification of LINC01320. This requires further study.

## Data Availability

All the data is available from the corresponding author due to reasonable request.

## Highlights

LINC01320 promotes the progression of gastric cancer;

LINC01320 sponges miR-495-5p in gastric cancer cells

miR-495-5p targets RAB19 in gastric cancer cells

LINC01320 is modified by METTL14 in gastric cancer cells
